# Synthesis of Copper Nanocluster and Its Application in Pollutant Analysis

**DOI:** 10.3390/bios11110424

**Published:** 2021-10-28

**Authors:** Yan Xue, Zehua Cheng, Mai Luo, Hao Hu, Chenglai Xia

**Affiliations:** 1 State Key Laboratory of Quality Research in Chinese Medicine, Institute of Chinese Medical Sciences, University of Macau, Macau 999078, China; yc17538@um.edu.mo (Y.X.); yb87516@um.edu.mo (Z.C.); yc07547@um.edu.mo (M.L.); 2 Affiliated Foshan Maternity & Child Healthcare Hospital, Southern Medical University, Foshan 528000, China; 3 School of Pharmaceutical Sciences, Southern Medical University, Guangzhou 510150, China

**Keywords:** sensor, fluorescence, pollutant, nanocluster, environmental analysis, pesticide, heavy metal, sulfide, explosive

## Abstract

Copper nanoclusters (Cu NCs) with their inherent optical and chemical advantages have gained increasing attention as a kind of novel material that possesses great potential, primarily in the use of contaminants sensing and bio-imaging. With a focus on environmental safety, this article comprehensively reviews the recent advances of Cu NCs in the application of various contaminants, including pesticide residues, heavy metal ions, sulfide ions and nitroaromatics. The common preparation methods and sensing mechanisms are summarized. The typical high-quality sensing probes based on Cu NCs towards various target contaminants are presented; additionally, the challenges and future perspectives in the development and application of Cu NCs in monitoring and analyzing environmental pollutants are discussed.

## 1. Introduction

Metal nanoclusters (MNCs) with ultra-small and tunable sizes, excellent photoluminescent efficiency, long fluorescence lifespan, desirable physical and biochemical stability and relatively low toxicity, have prompted the great advancement of research in both theoretical and practical fields [1,2]. The last decade witnessed the successful synthesis of novel MNCs and their applications in fluorescent sensors mainly based on gold (Au) and silver (Ag) nanoclusters. Meanwhile, copper nanoclusters (Cu NCs) have gradually gained increasing attention due to their chemical similarity with Ag NCs and Au NCs, distinct fluorescent characteristics, and in particular the low-cost and easier accessibility of their precursors as well as facile preparation procedures [3,4]. With the help of various functional ligands, it is possible to tune their emission wavelengths and obtain highly photoluminescent nanoclusters, providing potential for large-scale applications. More importantly, Cu NCs possess additional merits over other noble metal clusters with their excellent biocompatibility [5,6]. The fluorescent probes based on Cu NCs have demonstrated their versatility in sensing, lighting and bioimaging in clinical diagnosis and treatment [6,7,8,9]. Meanwhile, the significance of monitoring and analyzing various contaminants for the purpose of environmental safety should also be emphasized due to the widespread application of Cu NCs in this field.

In this review, we emphasize the recent progress of Cu NCs for application in environmental analysis. We first make a brief introduction of the common synthesis approaches of Cu NCs with a highlight on their intriguing optical properties. In the second section, we categorize the mainstream strategies based on Cu NCs in terms of sensing mechanisms. In the following part, we mainly present several typical novel Cu NCs targeting various contaminants in the environment, including pesticides, heavy metals, sulfide anions, as well as aromatic compounds. In the end, we conclude the article by discussing the challenges and prospects in the future development of Cu NCs as sensors for environmental pollutants.

## 2. Preparation Methods and Sensing Mechanism of Cu NCs

### 2.1. Preparation Methods

#### 2.1.1. Blue Emission

**Scheme 1:**
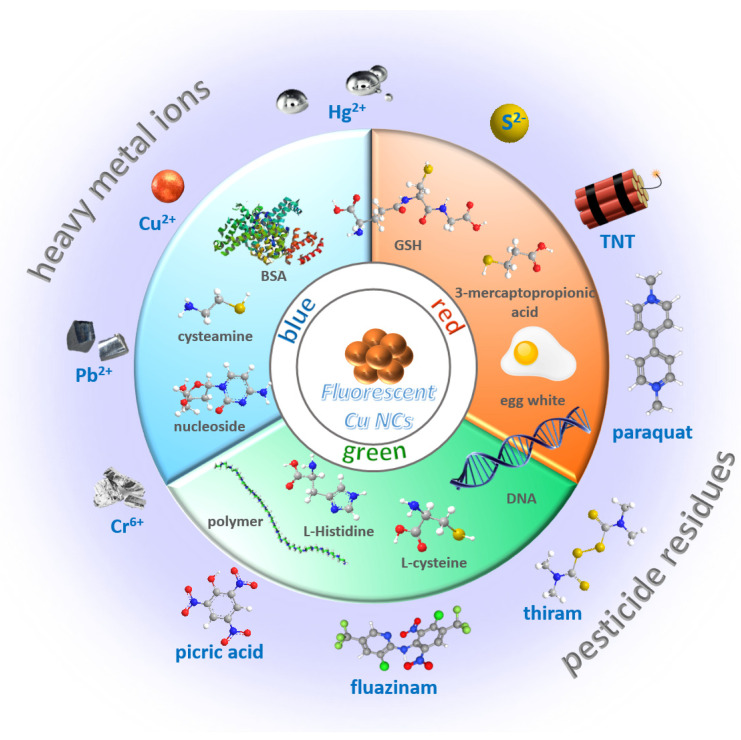
Summary of ligands and templates for Cu NCs and their typical applications.

In the recent decade, various fluorescent Cu NCs with a wide range of emission wavelengths from blue to red were developed with employment of different ligands and templates (Scheme 1). Cu NCs with blue emitting fluorescence have been covered most frequently in the latest research [7,10,11,12,13]. They are reported to have a broad range of applications, including detection of various contaminants and bioimaging [9,14,15,16,17]. The first blue emitting Cu NCs were reported in 2011 with bovine serum albumin (BSA) as template [18]. This novel type of nanocluster was synthesized in a one-pot method by using CuSO_4_ and BSA aqueous solution in the presence of NaOH. The as-prepared Cu NCs showed luminescence emission maxima at 410 nm with a high quantum yield of 15%. MALDI-TOF mass spectrometry was utilized to determine the precise atom number of the copper core. And the composition of this Cu NCs include Cu_5_ and Cu_13_. Since BSA is a very popular protein template in the synthesis of metal nanoclusters, many other BSA-Cu NCs with blue luminescence were subsequently reported [12,19,20]. Apart from proteins, thiolate ligands were also used widely to prepare Cu NCs [17,21,22,23,24]. In this category, glutathione (GSH) was a typical one [25,26,27]. In a study, Cu NCs with strong blue fluorescence were synthesized with GSH as both stabilizing and reducing agent [13]. These GSH-Cu NCs exhibit good photostability and storage stability with a quantum yield of 10.6%. Yang’s group reported their successful fabrication of Cu NCs stabilized with L-cysteine for the first time in a simple and green method. The most notable advantage of this material is that the L-cysteine-capped Cu NCs could be stored for more than 3 months in powder form without perceptible changes in their optical property. Moreover, a series of bi-ligand Cu NCs were developed using both thiosalicylic acid and cysteamine as protective and reducing agents [21]. The quantum yields of the products varied from 18.9% to 34.0% with the increase in cysteamine portion. This phenomenon results from the electron transfer to copper inducted by cysteamine’s terminal amine. Simultaneously, the thiosalicylic acid ligand absorbs energy through aromatic ring. Thus, more energy was released in the case of a higher cysteamine ratio. Interestingly, a pH-dependent synthesis method was reported that could produce Cu NCs with blue or yellow fluorescence emission under basic or acidic environments [28]. The two nanoclusters were obtained with the same method using trypsin as template and hydrazine hydrate as a reducing agent. The difference in pH value influences the conformational states of trypsin, and consequently results in the formation of Cu NCs with different sizes and fluorescent properties. And the fluorescence intensity of the trypsin-templated Cu NCs was further improved by adjustment of several synthesis conditions, such as molar ratio of CuSO_4_/N_2_H_4_, reaction temperature and reaction time. A pH-dependent Cu NC was synthesized in the presence of lysozyme and NaBH_4_. The Cu NCs showed potential in pH sensing since its fluorescence intensity increases with the decrease in pH [29]. In another study, a bright-blue Cu NCs was prepared with lysozyme as stabilizer and N_2_H_4_ as reducing agent. The Cu NCs were pH-stable and successfully applied in Hela cell labeling [30].

#### 2.1.2. Green Emission

Green emitting Cu/Ag bimetallic nanoclusters were synthesized by Mao et al. using DNA as template with the assistance of NaBH_4_ [31]. In this work, a series of DNA-Ag NCs and DNA-Cu/Ag NCs were prepared with several different single-stranded C-rich DNA templates. The fluorescence emission of the nanoclusters varies from green to orange. The study found that the nanoclusters with longer emission wavelengths tend to be constructed by templates with more consecutive cytosine bases. Another research designed Cu NCs protected by a polymer via a special UV-light-induced synthesis approach [32]. The synthesis procedure was simple and facile. Cu NCs were obtained through reduction of Cu(NO_3_)_2_ with ascorbic acid (AA) in the presence of polyethyleneimine (PEI) under UV light irradiation. Compared with stirring method, the PEI-Cu NCs prepared by UV-light-induced approach displayed higher quantum yield and significantly enhanced fluorescence intensity. By the means of UV-light irradiation, the absolute quantum yield of PEI-Cu NCs was raised from 0.98% to 2.63% and its fluorescence intensity increased 3.7 times. Additionally, some amino acid ligands such as cysteine and histidine were also employed in the development of green emitting Cu NCs [33,34]. In an intriguing case, pH-stimulus-responsive Cu NCs were fabricated using L-cysteine and chitosan as ligands [35]. The as-prepared Cu NCs exhibited orange-red luminescence emission at pH 4.5, and showed cyan-green emission with aggregation-induced emission (AIE) behavior when the pH was above 7.4. The special phenomenon results from the weakening of H-bonding between carboxylic group of cysteine and the protonated amine moiety of chitosan caused by pH adjustment to 7.4. The stable overall environment around the cluster core was disrupted, leading to the formation of aggregates. Furthermore, the change in interatomic bond distance in the aggregation state brought about a blue shift of emission, and as a result, green luminescence emission was generated.

#### 2.1.3. Orange/Red Emission

Many research groups developed GSH capped Cu NCs with orange or red fluorescence emission [36,37,38]. A one-pot sonochemical synthesis method was established for the preparation of GSH-Cu NCs by Wang et al. [39]. In this method, Cu (NO_3_)_2_ and GSH were mixed in water by 1: 4 ratio, and the pH of solution was adjusted to 6 with NaOH. The reaction was conducted in 15 min via ultrasonic irradiation, which is facile, fast and easy to operate. The as-synthesized GSH-Cu NCs exhibits bright red luminescence at λ_max_ = 606 nm and quantum yield up to 5.3%. As mentioned above, there are also many fluorescent GSH-Cu NCs that have been reported with blue emission. The main difference in the synthesis process is that red emitting GSH-Cu NCs utilized GSH as both a stabilizing and a reducing agent in acidic condition, while blue emitting ones were synthesized in basic condition or reduced by AA. DNA template is also frequently exploited in the fabrication of Cu NCs with red emission [40,41,42,43]. Li et al. developed copper nanoclusters templated by poly(thymine)-DNA with a fluorescence emission of 627 nm [44]. The DNA-Cu NCs were produced by a facile reaction between copper sulfate and poly-T DNA in 3-(N-morpholino) propanesulfonic acid buffer with the help of sodium ascorbate. Intriguingly, a research group successfully synthesized Cu NCs with strong orange emission utilized egg white as template [45]. The formation of Cu NCs relies on the interaction between multiple functional groups in egg white and CuSO_4_. Hydrazine hydrate was employed as reducing agent and NaOH was used to provide the basic environment. The reaction proceeded extremely fast, since it is carried out under microwave.

#### 2.1.4. Near Infrared Emission

In recent years, some Cu NCs showing near infrared emission have also been developed and applied in fluorescent sensors. Li et al. reported novel Cu NCs using denatured bovine serum albumin (dBSA) as templates [46]. The reaction was carried out at room temperature with hydrazine hydrate as reducing agent. In particular, the denatured BSA could form Cu NCs more efficiently, since it possesses more free cysteine with –SH groups than BSA. The as-prepared nanoclusters were successfully utilized to detect heparin by aggregation-induced quenching mechanism. Another study synthesized a near infrared emitting copper nanocluster co-protected by thiolate and phosphine ligands [47]. The structure of this nanocluster is atomically precise with the formula of [Cu_15_(PPh_3_)_6_(PET)_13_]^2+^. The product shows bright photoluminescence at 720 nm with crystallization induced emission enhancement property. More importantly, this research established a controllable synthesis strategy for atomically precise Cu NCs, which is meaningful and instructive to further study of their optical properties.

### 2.2. Sensing Mechanisms

#### 2.2.1. Turn Off

On account of the prominent fluorescence property and cost-effectiveness of Cu NCs, numerous Cu NCs-based sensing probes have been developed for multitude of analytes. The majority of Cu NCs-based fluorescent sensors follow turn-off mechanism that the analytes are detected due to the decrease in fluorescence intensity.

Inner filter effect (IFE) is a most common strategy in the development of turn-off sensors [48,49,50,51]. The inner filter effect refers to the fluorescence quenching process that the quencher absorbs the emission or excitation light of fluorophore. A study in this context established a label-free assay for nitrofurantoin using adenosine-stabilized Cu NCs [16]. Nitrofurantoin’s UV absorption band located at 250–430 nm happens to overlap the Cu NCs’ fluorescence excitation and emission spectra. In this way, the excitation and emission light could be shielded by nitrofurantoin, leading to decrease in fluorescence intensity.

Some sensing methods for the detection of electron-rich species were constructed based on the electron transfer mechanism [44,52,53]. Cao et al. developed tannic acid (TA) capped Cu NCs that could selectively detect ferric ions [52]. Fe^3+^ could form donor-accepter complex with the TA on the surface of Cu NCs, leading to fluorescence quenching through electron transfer process, and the quenching obeys a static quenching mechanism since the fluorescence lifespan was independent of the concentration of Fe^3+^. The high selectivity of this sensor is related to the ferric ions’ electronic structure. Fe^3+^ has five half-filled d orbits so that it exhibits stronger electron-accepting ability than other common ions.

Another major cause of fluorescence quenching in turn-off sensing methods is aggregation-induced quenching, which is widely employed in analysis of metal ions [26,39,54]. A GSH-Cu NCs based probe for Hg^2+^ was constructed based on Hg^2+^-induced aggregation of the nanoclusters [13]. Hg^2+^ linked the Cu NCs through the interaction with GSH ligands, leading to fluorescence quenching. According to the zeta potential measurement results, the surface charge of GSH-Cu NCs became less negative in the presence of Hg^2+^, which destroyed the stability of Cu NCs and generatesd aggregates. The aggregation was further evidenced by increased diameter in TEM and enhanced light scattering signal. 

#### 2.2.2. Turn On

The main strategy for turn-on sensors is a signal off-on process that the fluorescence would be quenched in the first place and subsequently recovered via the addition of analyte. Surfactant-free copper nanoclusters were exploited as a turn-on probe for quantification of vitamin C [55]. In the first step, the fluorescence was quenched by the formation of Fe-Cu NCs conjugate through a static quenching mechanism. On account of the strong reducing property of vitamin C, the Fe^3+^ were converted into Fe^2+^, which consequently resulted in the dissociation of Fe-Cu NCs conjugate and restored fluorescent intensity.

As AIE has become an enticing topic in recent years, many turn-on sensors were developed inspired by this phenomenon. Boonmee et al. established a fluorescent assay based on Al^3+^ induced AIE of cysteamine-capped Cu NCs [56]. Al^3+^ could interact with the amino groups on the surface of Cu NCs, causing aggregation of nanoclusters. The detection was realized through fluorescence enhancement triggered by the aggregation. Similar strategy was also applied in detection of S^2−^ and Pb^2+^ [10,57]. Specially, some studies directly utilized the formation of copper nanoclusters as a selective Cu^2+^ turn-on sensing method [38,58]. Apart from AIE, confinement-induced enhanced emission (CIEE) is also a strategy that frequently used to provide highly fluorescent Cu NCs with high quantum yield [36,59,60]. The improved fluorescence property was achieved by restricting the free movement of fluorescent molecules with the host materials [61]. Yang et al. constructed a turn-on sensing method for bio-enzyme based on CIEE with ultra-high sensitivity [62]. In this work, a fluorescent composite GS-Cu NCs and LDH (GS-Cu NCs/LDH) was fabricated on the basis of surface confinement effect, affording excellent quantum yield, improved stability and long fluorescence lifetime. The surface confinement effect of this material was firstly inhibited by hyaluronic acid (HA). In the presence of hyaluronidase (HAase), the HA would be hydrolyzed, leading to the recovery of fluorescence enhancement. In this way, a fluorescent assay for cancer biomarker HAase was successfully developed.

#### 2.2.3. Ratiometric Analysis

Ratiometric fluorescence sensors have also attracted increasing research interest since it exhibits dominant advantages in accuracy, sensitivity and stability. In a ratiometric sensing approach, the detection is achieved by the intensity ratio of dual-emission peaks, which could eliminate the interference of environment and probe concentrations by self-calibration. A CuNC@AF660 sensor was fabricated for ratiometric sensing of calcium ions. The fluorescence intensity of Cu NCs emission peak was enhanced gradually with the increase in Ca^2+^ concentration through ion-induced AIE mechanism. The Alex Fluor 660 NHS ester fabricated on Cu NCs provides the inner-reference signal.

In some ratiometric sensing systems, one emission peak is quenched while the other is enhanced in the presence of analytes. In these cases, the underlying mechanism is usually ascribed to Förster resonance energy transfer (FRET). A probe based on aptamer-modified Copper @ Gold nanoclusters (apt-Cu@Au NCs) was designed for ratiometric detection of Hg^2+^ [63]. The mercury ions were captured by T-rich nucleic acid single strands on the surface of nanoclusters. The formation of T-Hg^2+^-T structure led to aggregation between the two fluorophores and affected the fluorescence intensity via FRET between Au NCs and Cu NCs.

## 3. Sensing Applications Based on Cu NCs

### 3.1. Pesticides as Target Analytes

Pesticide residues have been widespread in the environment due to the excessive use of pesticides in agricultural production and horticulture [64,65,66]. It has been found that even trace amounts of highly toxic pollutants could greatly threaten the ecosystem and human health. 

**Figure 1:**
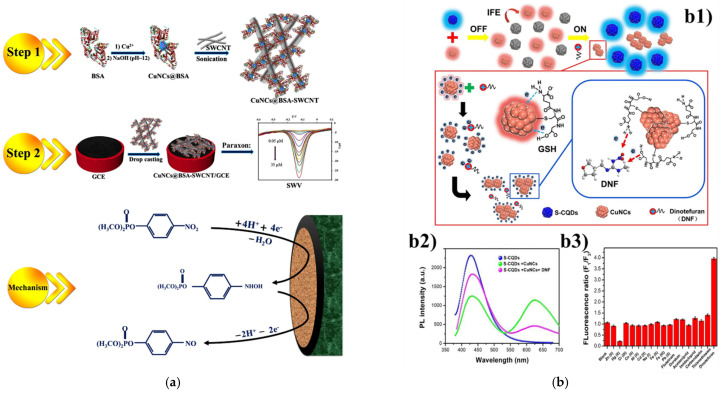
(**a**) Schematic representation of biosensor fabrication and suggested mechanism for paraoxon reduction; (**b**) (b1) The schematic illustration of ratiometric determination of dinotefuran based on S-CQDs/CuNCs probe; (b2) the emission spectra of S-CQDs and S-CQDs/CuNCs with and without the addition of DNF; (b3) selectivity analyses of as-developed ratiometric fluorescent probe mixed various interfering chemicals.

In this section, we mainly highlight the Cu NCs-based detection systems targeting common pesticides (Table 1), e.g., paraoxon, dinotefuran (DNF), o-phenylenediamine (OPD), dithiocarbamates (DTCs), thiram, paraquat, fluazina, nitrofurantoin (NFT). Copper nanocluster was employed in the construction of an enzyme-free electrochemical biosensor toward paraoxon as the model of organophosphates (OP) [12]. The biocompatible nanocomposite Cu NCs@BSA-SWCNT was synthesized by combining bovine serum albumin (BSA) template-capped Cu nanoclusters (Cu NCs@BSA) and single-walled carbon nanotubes (SWCNT), which demonstrated remarkable sensitivity and high electrocatalytic property toward the reduction of paraoxon (Figure 1a). The entrapped Cu NCs rendered high electrical conductivity and concentrated the redox active centers on the surface of the probe, while the SWCNT further enhanced the electrocatalytic activity along with conductivity of the glassy carbon electrode (GCE) surface. The linear range was 0.5–35 μM, with a limit of detection of 12.8 nM. Moreover, this electrochemical nanocomposite was found to be able to effectively determine paraoxon with satisfied recoveries ranged from 93% to 104% in a real water sample. In order to detect and monitor the residues of dinotefuran (DNF), which has been widely used in agriculture, novel sensing probes and platforms based on fluorescent copper nanoclusters have been constructed. Yang et al. [67] established a dual-emission ratiometric fluorescent probe by integrating sulfur-doped carbon quantum dots (S-CQDs) and Cu NCs with mixed fluorescent signals (Figure 1b). The as-developed hybrid (S-CQDs/Cu NCs) was observed to demonstrate desirable sensitivity and selectivity towards DNF with linear range from 10 to 500 μM. In this nanocomposite, IFE caused the decrease in fluorescent signals of S-CQDs with the addition of Cu NCs. In the presence of dinitefuran, the IFE between S-CQDs and Cu NCs would be weakened due to the aggregation of Cu NCs, leading to the restoration of S-CODs fluorescence. In the case of honey as the real sample, this ratiometric fluorescent S-CQDs/Cu NCs showed good analysis performance for the detection of DNF. Besides, another ratiometric detection system was proposed for o-phenylenediamine (OPD) based on the use of copper nanoclusters [27]. This method achieved signal amplification and ideal sensitivity through the combined influence of the oxidation reaction and FRET effect. With addition of OPD into Cu NCs, the fluorescence intensity of the Cu NCs at 432 nm decreased, while the oxidized OPD (oxOPD) showed strong fluorescence at 557 nm. This detection strategy was able to determine OPD in real water samples with an ultralow limit of detection of 0.096 g L^−1^. Furthermore, a rapid and sensitive detection method of dithiocarbamates (DTCs) with dual functionality in fluorescence and colorimetry was established utilizing CTAB -entrapped Cu NCs [68]. Owing to the fluorescence quenching of the Cu NCs with addition of DTCs, the detection system demonstrated remarkable sensitivity and selectivity toward DTCs with a linear range from 1 to 100 mg kg^−1^ and a low limit of detection of 0.63 mg kg^−1^.

**Figure 2:**
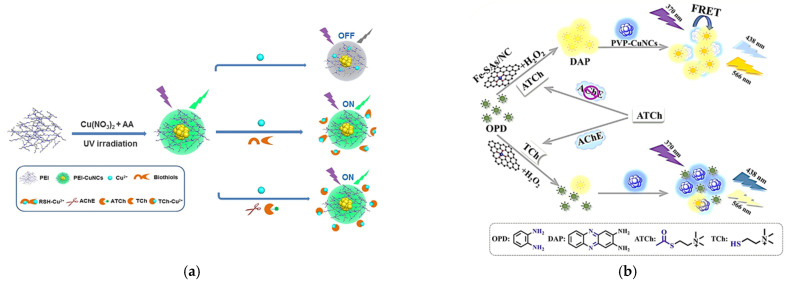
(**a**) Schematic illustration of Cu^2+^-quenched fluorescence turn-on assay for the detection of biothiols and AChE; (**b**) Schematic illustration of this ratio fluorescent sensing strategy for AChE activity sensing.

Acetylcholinesterase (AChE) as a key enzyme in relation to Alzheimer’s disease (AD) has been a hot detection target for many novel sensing probes based on copper nanoclusters. Due to the intrinsic mechanism of AChE inhibition in the presence of organophosphates, these strategies could be extended for pesticide detection. Yang et al. [32] reported the successful establishment of a fluorescence sensing method for the determination of multiple analytes through the induction of UV-light. The polyethyleneimine-protected copper nanoclusters (PEI-Cu NCs) presented strong stability and fluorescence intensity, which was employed in the development a label-free assay for sensitive detection of Cu^2+^ (Figure 2a). The interaction between Cu^2+^ with the -SH functional groups in biothiols generated the RSH-Cu^2+^ complex, which could trigger the fluorescence recovery of the PEI-Cu NCs. GSH and cysteine (Cy) as two biothiols were consequently detected within a linear range of 1–25 μM and 0.5–25 μM, respectively. In terms of the AChE activity, thiocholine (TCh) containing -SH group was produced with the hydrolyzation of acetylthiocholine by AChE. TCh interacted with Cu^2+^ to generate TCh-Cu^2+^ complex, which triggered the fluorescence recovery of the PEI-Cu NCs. The response toward AChE was linear ranging from 3–200 UL^−1^ with a limit of detection of 1.38 UL^−1^. Furthermore, this sensing platform was also employed in the detection of tacrine, a typical inhibitor of AChE with the IC_50_ value at 53.4 nM. A novel ratiometric florescence probe toward the evaluation of AChE activity was established through incorporating single-atom nanozyme (SAzyme) with the polyvinylpyrrolidone-protected copper nanocluster (PVP-Cu NCs) [14]. The SAzyme was first achieved through conjugating single-atom iron with N-doped porous carbon (Fe-SAs/NC) with peroxidase-like activity (Figure 2b). The obtained Fe-SAs/NC was observed to catalyze oxidation of OPD to 2,3-diaminophenazine (DAP) in the presence of H_2_O_2_. The fluorescent DAP displayed fluorescence emission at 566 nm, while the PVP-Cu NCs exhibited fluorescence emission peak at 438 nm. With the increase in the DAP concentration, the fluorescence intensity of PVP-Cu NCs was found to be quenched. The mechanism behind the quenching effect was confirmed to be FRET between PVP-Cu NCs and DAP through the measurement of fluorescence lifetime. This dual-emission signal system demonstrated high sensitivity toward AChE activity with a linear range between 2–70 UL^−1^ and a low limit of detection at 0.56 UL^−1^. Although this exploration proved the effectiveness of SAzymes in AChE analysis, the further investigation into its feasibility in its inhibitor evaluation, especially pesticide residues, is still urgent. Based on the IFE, another sensitive analytical method was proposed based on 5′-Dithiobis-(2-nitrobenzoic acid) (DTNB) and copper/silver nanoclusters [34]. This method exhibited high sensitivity with strong fluorescence within the linear range between 0.1–1.0 U L^−1^ and with a very low detection limit of 0.03 UL^−1^. Furthermore, the sensor constructed in this work showed distinct color change that could be distinguished by naked eyes, which further improved its feasibility in detecting AChE from other substance selectively.

### 3.2. Heavy Metals as Target Analytes

Heavy metals are notorious and hazardous contaminants in environment due to their low degradability, acute toxicity, high bioaccumulation, and other factors. Herein, we summarized nanoprobes based on Cu NCs for the determination of several representative heavy metal ions including mercury ions (Hg^2+^), lead ions (Pb^2+^), chromate anions (Cr^6+^) and copper ions (Cu^2+^) (Table 2). 

#### 3.2.1. Mercury Ions

**Figure 3:**
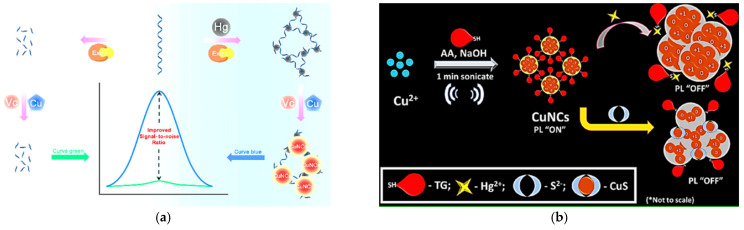
(**a**) Schematic Illustration of Hg^2+^ Quantification Based on Fluorescence Regulation of CuNCs via DNA Template Manipulation; (**b**) Schematic representation for the possible mechanism of Hg^2+^ and S^2−^ detection using TG-CuNCs.

**Figure 4:**
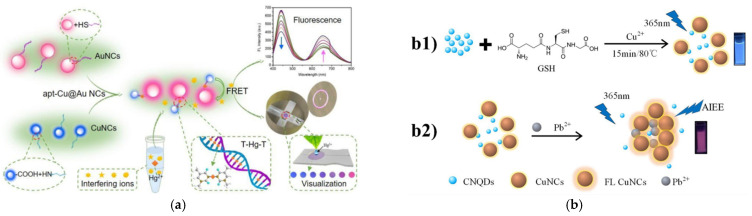
(**a**) Schematic of apt-Cu@Au NCs aptasensor for Hg^2+^ ions detection; (**b**) (b1) Schematic of the preparation of CuNCs–CNQDs nanohybrid; (b2) Schematic for the Pb^2+^ detection assay.

Mercury has been found to be prevalent in the food chain, which poses a huge threat to the health of the ecosystem and humans. The conventional monitoring methods to Hg^2+^ could barely satisfy the soaring demand of rapid, facile and sensitive determination of this substrate in real samples [70,71,72]. An analytical method with high signal-to-noise ratio was constructed by regulating the fluorescence of copper nanoclusters through engineering the reticular poly(T) DNA template [40]. The fluorescence intensity of as-designed copper-based nanoclusters was observed to be enhanced to a great extent by using this DNA template with its unique structure and its resistance towards enzyme digestion. This novel DNA-templated nanomaterial exhibited excellent sensitivity and selectivity toward mercury ions with a desirably linear range from 50 pM to 500 μM and a remarkably low limit of detection at 16 pM (Figure 3a). Meanwhile, owning to the interplay between Hg^2+^ and GSH, this sensory probe could also be employed to detect the inhibition of GSH by Hg^2+^ and thus to determine the GSH in real biological samples. In the real sample analysis, this biosensor was found to be a reliable and feasible analytical method. An instant-response and facile analysis platform targeting Hg^2+^ and S^2−^ ions was established through incorporating copper nanoclusters with 1-Thio-β-Dglucose as a capping ligand via ultrasonication, the synthesis of which could be completed in as fast as one minute [73]. As shown in Figure 3b, the as-synthesized TG-Cu NCs demonstrated the emission maximum at 430 nm, and through fluorescence quenching, this probe is able to detect Hg^2+^ and S^2−^ ions with very low detection limits at 1.7 and 1.02 nM, respectively. This on-site detection and monitoring of the target analytes in real samples (e.g., tap, river and pond water) could be achieved easily with the developed cheap smartphone-aided and paper-based kit. Shi et al. [63] developed a ratiometric sensing strategy toward mercury ions by modifying copper nanoclusters @ gold nanoclusters via aptamer (apt-Cu@Au NCs). The changes in the fluorescence of this novel probe is attributed to the FRET strategy, which was achieved through the thymidine–Hg–thymidine (T–Hg–T) structure generated by addition of Hg^2+^ into the solution containing apt-Cu@Au NCs. Hg^2+^ ions could be detected with a range from 0.1 to 9.0 μM with the detection limit of 4.92 nM (Figure 4a). Meanwhile, to improve the feasibility, practicality, cost-effectiveness and the user-friendliness of this sensor, a smartphone-aided visualization method based on the fluorescent microfluidic chip was proposed to enable the facile and on-site determination of the target analytes. This sensing platform exhibited good performance in real samples with recovery rate at 101.83–114.00%. In another Hg^2+^ sensor, Cu NCs were assembled with polymers with multi-thiol groups as templates (Cu NCs@P-8B) through the simple one-pot approach [74]. This nanohybrid demonstrated satisfactory water stability, high sensitivity and responsiveness to pH and temperature, with a linear range between 10–100 μM and a limit of detection of 10 μM. Its performance in the real sample test with human urine indicated its potential in practical applications. 

#### 3.2.2. Lead Ions

Similarly, numerous novel probes have also been developed and investigated to monitor lead ions (Pb^2+^) in the environment and in the drinking water system, as lead is highly biotoxic and threatens the health of children, in particular. Intake of extremely low levels of lead can cause cardiovascular, nervous, and reproductive diseases [75,76]. Based on the nanocomposite of copper nanoclusters and carbon quantum dots (Cu NCs–CNQDs) blended in nitrogen, a novel ratiometric fluorescence determination method was reported to detect and analyze the trace amount of hypertoxic lead ions in a popular aquatic product porphyra [77]. As illustrated in Figure 4b, the fluorescence emission of the Cu NCs-CNQDs was observed to peak at 468 nm and 632 nm. The fluorescence of this Cu NCs was considerably enhanced when Pb^2+^ was added into the solution owing to the aggregation-induced emission enhancement (AIEE) between Pb^2+^ and Cu NCs. Meanwhile, the fluorescence of CNQDs remained the same, which could be taken as the self-calibration signal, and the Cu NCs-CNQDs demonstrated remarkable sensitivity and selectivity toward Pb^2+^ in porphyra ranging from 0.010 mg/L to 2.5 mg/L, with a low detection limit of 0.0031 mgL^−1^. Goswami and co-workers [18] proposed a BSA-capped Cu quantum clusters (CuQC@BSA) as the probe targeting Pb^2+^. The as-developed nanocomposite was highly sensitive toward Pb^2+^, which is attributed to the fluorescence quenching effect in ultra-low concentration of hydrogen peroxide at 200 ppm. It also performed well, even in the presence of interference from other metal ions. In another work, GSH-stabilized Cu NCs (GSH-Cu NCs) was fabricated successfully via the simple sonochemical synthesis method [39]. Based on the fluorescence quenching mechanism, this hybrid showed excellent performance in the label-free detection of Pb^2+^ with a linear range between 1 and 160 nM and a detection limit as low as 1.0 nM.

#### 3.2.3. Chromate Anions

**Figure 5:**
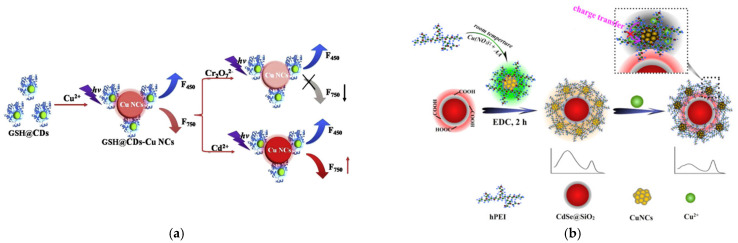
(**a**) Schematic illustration of the preparation of GSH@CDs-Cu NCs and the application in the detection of Cr_2_O_7_^2−^ and Cd^2+^ ions; (**b**) Schematic illustration of the preparation of dual-emission ratiometric fluorescence probe and its sensing mechanism to Cu^2+^ ions.

Chromium (Cr (VI)) has been extensively used in the modern industrial production and has also been a common contaminant in the environment. Considering its hugely harmful effect on human health, various analytical methods for the detection of chromium have been designed and, similar to other heavy metal contaminants, many novel sensors based on metal nanoclusters for Cr (VI) have been constructed. Bai and coworkers [78] proposed a ratiometric fluorescent probe for the convenient and effective detection of Cr_2_O_7_^2−^ or Cd^2+^ by integrating GSH-based carbon dots with copper nanoclusters. As shown in Figure 5a, the carbon dots-stabilized copper nanoclusters (GSH@CDs-Cu NCs) exhibited two obvious emission peaks at 450 nm and 750 nm, respectively. Owing to fluorescence quenching or enhancement of the nanohybrid, the GSH@CDs-Cu NCs showed good sensitivity and selectivity toward Cr_2_O_7_^2−^ with a linear range from 2 to 40 μmol L^−1^ and a detection limit of 0.9 μmol L^−1^, and Cd^2+^ with a linear range from 0 to 20 μmol L^−1^ and a detection limit of 0.6 μmol L^−1^. In addition, successful application of the fluorescence test strips was achieved in the rapid detection of Cr_2_O_7_^2−^ ions with distinct fluorescent color changes from pink to purple under UV light. In the test of real samples, the recovery rates of the target analytes in various water samples were in the range from 102% to 109% with the relative standard deviations (RSD) smaller than 4.5%. And the recovery rates of the target analytes ranged from 97%to 108% with the RSD smaller than 3.5%. Through the one-pot galvanic reduction approach, gold-copper nanoclusters capped by cysteamine (CA-AuCu NCs) were prepared for the detection of chromium(VI) and dopamine, the levels of which are critical to the development of severe diseases [23]. The distinct optical properties of this probe made it an ideal candidate in the determination of the target analytes in real samples, such as tap water, lake water, sea water and human urine. In particular, this switch-off probe was responsive in the linear range of 0.2–100 μm for Cr (VI) and with a detection limit of 80 nM.

#### 3.2.4. Copper Ions

The prevalence of copper ion residues in the food and in the environment is another looming danger due to damage to the human liver and the nervous system caused by excessive Cu^2+^. Typically, Alzheimer’s disease (AD), as a common neurodegenerative disease, might be caused by excessive accumulation of copper ion and corresponding oxidative stress [79,80]. Hyperbranched polyethyleneimine-protected copper nanoclusters (hPEI-Cu NCs) were integrated with the silica-coated CdSe quantum dots (QDs) to obtain a novel ratiometric and visual analytical probe toward copper ions through the simple and green one-pot chemical reduction method at room temperature [81]. This QDs@Cu NCs probe exhibited high sensitivity and visual detection capability toward Cu^2+^, which could be attributed to the self-calibration function of the dual-emission fluorescence signals inherent in the ratio-based fluorescence probe. The fluorescence of hPEI-Cu NCs was quenched due to the interaction of its amine groups with Cu^2+^, while the fluorescence intensity of QDs remained unchanged, and the distinct color changes could be easily observed by the naked eye under the UV-light. The linear range of the probe in Cu^2+^ concentrations was, approximately, from 22 nM to 8.8 μM, with an estimated detection limit of 8.9 nM (Figure 5b). When applied in the real water samples with different concentrations of Cu^2+^, the as-synthesized probe exhibited satisfactory recovery. 

### 3.3. Sulfide as Target Analytes

**Figure 6:**
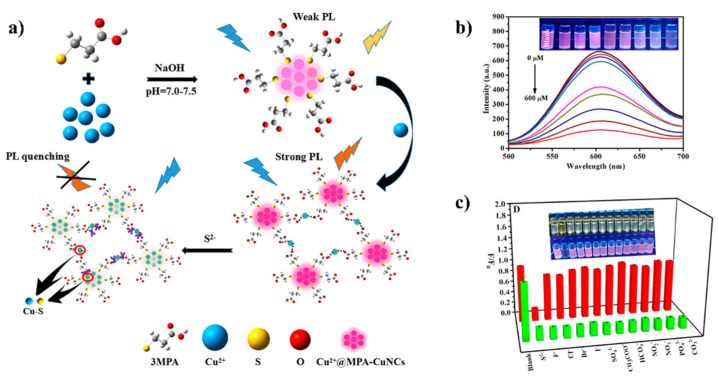
(**a**) Schematic illustration of the preparation of MPA-Capped Cu NCs and the aggregation induced PL weakening of Cu^2+^@MPA-Cu NCs in the presence of S^2−^; (**b**) fluorescence emission spectra of Cu^2+^@MPA-Cu NCs in the presence of various concentrations of S^2−^ in Tris-HCl buffer solution; (**c**) effect of different anions on the fluorescence intensity of Cu^2+^@MPA-Cu NCs (red pillars), and the influence on the sensor in the presence of different anions coexisting with S^2−^ ions (green pillars).

Another main category of environmental pollutants is sulfide ions, which are also by-products of massive industrial development. Their wide distribution in different formats (i.e., H_2_S, HS^−^ and S^2−^) in nature makes them a latent crisis, threatening human health. Cu NCs have also been extensively utilized to determine sulfide ions (Table 3). A “turn-on” determination strategy toward sulfide ion (S^2−^) was developed based on fluorescent copper nanoclusters with natural silk fibroin (SF) protein as template (SF@Cu NCs), which was synthesized via utilizing the environmental friendly and user-friendly one-pot method [10]. With the addition of S^2−^ ions into the aqueous solutions, the fluorescence intensity of the probe was observed to be increased, due to the inherent feature of aggregation/assembly-induced emission enhancement (AIEE). The S^2−^ could induce the self-assembly of copper nanoclusters into large rod-shape nanoparticles. This SF@Cu NCs nanohybrid demonstrated good sensitivity and selectivity toward S^2−^ with a linear range of 5.0 μM–110.0 μM and the limit of detection at 0.286 μM. In the real sample test, this analytical approach was found to be powerful in determining the sulfide ions at different concentrations with a satisfactory recovery rate of 95.6–101.6%. Another sensing platform for the determination of sulfide ions was reported. The main component of this approach was synthesized through modifying copper nanoclusters by 3-mercaptopropionic acid and with AIE induced by Cu^2+^ (Cu^2+^@MPA-Cu NCs) [92]. The preparation process of this luminescence probe was easy-to-operate and facile. The probe exhibited good linear detection of S^2−^ in the range from 0 to 600 μM, with a detection limit of 26.3 nM (Figure 6). In the quantitative detection of S^2−^, the Cu^2+^@MPA-Cu NCs sensor presented high selectivity and good accuracy in the simulated real environment, with recovery rates ranging from 92.5% to 104.5% and RSD less than 4%. Ratio-based sensing systems for the monitoring of sulfide ions have also been established based on all Cu NCs (G-R-Cu NCs) [93]. The fluorescent probe was obtained through rapid and simple synthesis procedure and exhibited remarkable selectivity and sensitivity in the real sample. 

### 3.4. Others 

**Figure 7:**
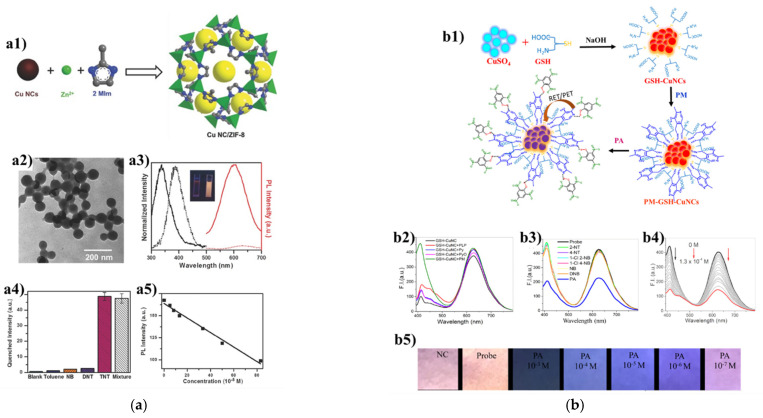
(**a**) (a1) Schematic illustration of the synthesis of Cu NC/ZIF-8 composites from Zn^2+^ ions, 2 Mlm and Cu NCs; (a2) TEM images of Cu NC/ZIF-8 composites; (a3) PL (red, excited at 365 nm) and PLE (black, detection wavelength 600 nm) spectra of Cu NCs (dotted lines) and Cu NC/ZIF-8 composites (solid lines); (a4) the decrease in PL intensity of Cu NC/ZIF-8 composites to blank, toluene, NB, DNT, TNT, and the mixture of all above (concentration of all compounds 500 × 10^−6^ M); (a5) Linear relationship between the PL intensity of Cu NC/ZIF-8 composites and the concentration of TNT; (**b**) (b1) Schematic representation for the synthesis of GSH-CuNCs and its interaction with PM and followed by with PA; (b2) fluorescent spectral changes of GSH-CuNCs upon addition of various vitamin B6 cofactors; (b3) fluorescence spectral changes of PM-GSH-CuNCs in the presence of various nitro-aromatics; (b4) fluorescence spectral changes of PM-GSH-CuNCs upon addition of increasing [PA]; (b5) fluorescence color changes of the modified paper strips upon interaction with different concentrations of PA (1 mM to 10^−7^ M) observed under UV light at 365 nm.

Cu NCs with their fascinating fluorescence properties as well as desirable stability have also been widely employed as sensing probes to determine aromatic compounds (Table 4). Trinitrotoluene (TNT), a popular explosive commonly used in military work, is indeed a dangerous contaminant in water. For the detection of TNT, a luminescent sensor was established by embedding copper nanoclusters (Cu NCs) into metal-organic frameworks (MOF) ZIF-8 [36]. Thanks to the confinement-assisted emission enhancement similar to AIE of fluorescence, the stability and emission intensity of the composite was largely improved, with an orange-color emission peak at 600 nm (Figure 7a). This Cu NC/ZIF-8 hybrid exhibited capability of detecting trace amounts of TNT, since it was selectively quenched in the presence of TNT. Similarly, there has also been an urgent need for the facile and sensitive detection of picric acid (2,4,6- trinitrophenol, PA). Patel et al. [37] reported an analytical system for PA based on the fluorescence quenching of glutathione stabilized copper nanoclusters and a vitamin B6 cofactor pyridoxamine (PM-GSH-Cu NCs) (Figure 7b). This nanoprobe exhibited excellent sensitivity and selectivity to PA in different solutions and real samples including tap water, river water and matchstick. To be specific, it could detect PA down to 27.4 × 10^−7^ M, and the paper strips based on this nanoprobe could determine PA with the naked eye at as low as 1 μM. 

Artificial food coloring has contributed greatly to the food manufacturing industry to increase the attractiveness of food by vivid colors. Due to the looming danger and risks to human health, some artificial colorants are banned from being added in food, while the use of others in food are strictly regulated. Quinoline yellow (QY) belongs to the one of the above two categories in various countries that ban colorants and strictly regulate substances. Analytical methods based on Cu NCs have been thus developed toward quinoline yellow in food products. Sivasankaran et al. reported their successful construction of a fluorescent-based probe for QY [97]. Owing to the quenching effect, the blue-fluorescence-emitting-L-cysteine stabilized Cu NCs (L-Cys-Cu NCs) demonstrated excellent sensitivity and selectivity toward QY with a low detection limit of 0.11 μM and a linear concentration range between 5.50 and 0.20 μM. Cu NCs-based sensing probes or systems have also been used in detection and monitoring of overuse or abuse of broad-spectrum antibiotics. An ultrasensitive biosensor based on glutathione-capped copper nanoclusters (GSH-Cu NCs) was designed to determine the trace level of furazolidone in the environment and control the overuse [98]. The blue-emitting fluorescent probe was prepared in a very short time. Due to the inner filter effect and static quenching between GSH-Cu NCs and furazolidone, the as-prepared probe exhibited a favorable linear range from 0.05 to 60 μM and a low detection limit at 0.012 μM. Furthermore, Cu NCs have also proven effectiveness in the detection of residues of 2,6-pyridinedicarboxylic acid (DPA) in the environment, which is notorious for its hazards toward human health and safety, as well as in vulnerable ecosystems. A portable sensing strategy was developed based on the Cu NC fluorescent test strip. The as-fabricated ratiometric sensing probe (GSH-Cu NCs/Eu^3+^) demonstrated its sensitivity, timeliness and convenience in determining DPA in the complicated solution with an ultra-low detection limit of 8 nM [99]. 

## 4. Conclusions and Perspectives

This review summarized and reported the latest development of fluorescent copper nanoclusters utilized in monitoring various types of environmental contaminants. Despite the fascinating advances, there is still a long and arduous way ahead in further improving Cu NCs to make them ideal materials for pollutant detection, quantitative determination, and, even, decomposition and removal. Firstly, the sizes of the current Cu NCs designed fall into a rather wide range, and the desirable optical properties of most Cu NCs heavily rely on excitation. Both of these two aspects may largely constrain the possibility of efficient application of Cu NCs in environmental analysis on a large scale. Secondly, efforts are imperative to further enhance the chemical and optical stability, as well as quantum yield of Cu NCs, so as to improve their feasibility as sensors in more complex, real environments, even with complicated, interfering factors. Lastly, the functions of the current probes are expected to be extended from pure detection of environmental pollutants to simultaneous detection, degradation and, even, removal. 

## Figures and Tables

**Table 1:** List of fluorescent probes based on Cu NCs for pesticide detection.

No	Analytes	Sensors	Ex./Em. Maxima (nm)	Sensing Mechanism	Linear Range	Limit of Detection (LOD)	Real Sample	Ref.
1	Paraoxon	Cu NCs @ BSA-SWCNT/GCE	325/420	electrochemical method	0.05–0.5 μM 0.5–35 μM	12.8 nM	water	[12]
2	Thiram Paraquat	Egg white- Cu NCs	344/600	turn off	0.5–1000 μM 0.2–1000 μM	70 nM 49 nM	water	[45]
3	Metham sodium	CTAB-Cu NCs	254/620	fluorescent based colorimetric method	1–100 mg kg^−1^	0.63 mg kg^−1^	apple, pear and cherry tomato	[68]
4	Fluazina	L-Cys-Cu NCs	365/497	turn off	0.05–25 µM	1.4 nM	pears and cabbage	[33]
5	o-Phenylenediamine	GSH-Cu NCs	334/432	ratiometric	0.15–110 μg L^−1^	93 ng L^−1^	industry water	[27]
6	Nitrofurantoin	Adenosine-Cu NCs	285/417	turn off	0.05–4.0 μM	30 nM	lake water	[16]
7	Dinotefuran	S-CQDs/Cu NCs	330/430	ratiometric	10–500 μM	7.04 μM	honey	[67]
8	AChE Methamidophos	DNA-Cu/Ag NCs	480/565	turn off turn on	0.05–2.0 U L^−1^ —	0.05 UL^−1^ 0.075 mg L^−1^ (IC_50_)	water and vegetable	[69]
10	AChE	L-His-Cu/Ag NCs	390/485	turn off	0.1–1.0 UL^−1^ and 1.0–7.0 UL^−1^	0.03 UL^−1^	—	[34]
9	AChE	PVP-Cu NCs	370/438	ratiometric	2.0–70 UL^−1^	0.56 UL^−1^	human serum sample	[14]
11	AChE	PEI-Cu NCs	365/495	turn on	3–200 UL^−1^	1.38 UL^−1^	human serum sample	[32]

**Table 2:** List of fluorescent probes based on Cu NCs for heavy metal detection.

No	Analytes	Sensors	Ex./Em. Maxima (nm)	Sensing Mechanism	Linear Range	Limit of Detection (LOD)	Real Sample	Ref.
1	Hg^2+^	Cu NCs@P-8B	400/535	turn off	10–100 μM	10 μM	aqueous solution	[74]
2	Hg^2+^	Curcuminoids-Cu NCs	350/440	turn off	0.5 nM–25 µM	0.12 nM	water	[54]
3	Hg^2+^	Cu NCs	340/560	turn off	2–40 μM	23 nM	water	[72]
4	Hg^2+^+	Trypsin-Cu NCs	360/567	turn off	0.1−100 μM	30 nM	human urine and serum samples	[28]
5	Hg^2+^	TdT-INAA-DNA-Cu NCs	343/600	turn off	0.2–500 nM	76 pM	environmental water	[41]
6	Hg^2+^	GSH–Cu NCs	360/445	turn off	10 nM–10 μM	3.3 nM	water and rice	[13]
7	Hg^2+^	poly(30T) DNA-Cu NCs	340/650	turn on	50 pM–2.5 μM and 2.5–500 μM	16 pM	lake water	[40]
8	Hg^2+^	DTT-Cu NCs/CNNS nanocomposite	395/615	electrochemiluminescence	0.05–10 nM	0.01 nM	lake and tap water	[71]
9	Hg^2+^	Metallothionein–Cu NCs	—	UV-VIS	97 nm–2.3 μM and 3.1–15.6 μM	43.8 nM	environmental water	[82]
10	Hg^2+^	GSH-Cu NCs	375/440	turn off	0.04−60 μM	22 nM	water	[26]
11	Hg^2+^	Cytosine rich- ssDNA-Cu/Ag NCs	470/550	turn off	40–550 nM	2.4 nM	lake and tap water	[31]
12	Hg^2+^	apt-Cu@Au NCs	470/656	ratiometric	0.1–9.0 μM	4.92 nM	porphyra	[63]
13	Hg^2+^	4-chlorothiophenol-Cu NCs	330/605	turn off	1–500 nM	0.3 nM	environmental water	[83]
14	Hg^2+^	BSA-Cu NCs	320/420	turn off	0.01 nM–10 μM	4.7 pM	water	[19]
15	Hg^2+^	BSA-Cu NCs	395/645	turn off	20–1000 nM	0.2 nM	—	[84]
16	Hg^2+^	L-Cys-Cu NCs	375/480	turn off	0.1–1000 μM	24 nM	human urine sample	[17]
17	Hg^2+^	dsDNA-Cu NCs	570/595	turn off	0.04−8 nM	4 pM	water	[70]
18	Hg^2+^	Ag/Cu NCs	colorimetric	turn on	0.1–700 nM	0.05 nM	aqueous sample	[85]
19	Hg^2+^	CDs-CuNCs	345/430,647	ratiometric	0–4000 nM	0.31 nM	Tap, lake water	[86]
20	Hg^2+^	BSA-Cu NCs/ BSA-Au NCs	365/398,616	ratiometric	0.06–1 µM and 1–4 µM	19.4 nM	Tap, mineral, lake water	[87]
21	Pb^2+^	BSA-Cu NCs	324/401 (fluorescent); 324/396 (light scattering)	turn off; turn on	30–180 nM; 3–21 nM	10 nM; 1 nM	environmental water	[88]
22	Pb^2+^	BSA-Cu NCs	325/410	turn off	0–200 ppm	—	—	[18]
23	Pb^2+^	GSH-Cu NCs	360/607	turn on	200–700 μM	106 μM	water	[57]
24	Pb^2+^	Cu NCs-CNQDs	365/468, 632	ratiometric	0.01–2.5 mg L^−1^	0.0031 mg L^−1^	porphyra	[77]
25	Pb^2+^	Metallothionein–Cu NCs	—	UV-VIS	0.7–96 μM	142 nM	environmental water	[82]
26	Pb^2+^	dsDNA-Cu NCs	340/605	turn off	0–150 pM	5.2 pM	tap water	[43]
27	Pb^2+^	GSH-Cu NCs	420/606	turn off	1–160 nM	1 nM	—	[39]
28	Pb^2+^	Cu NASs	340/590	turn off	2–100 nM	0.75 nM	aqueous sample	[89]
29	Cr_2_O_7_^2−^	GSH@CDs-Cu NCs	360/450,750	ratiometric	0–20 μM	0.9 μM	tap water, spring water samples and human urine	[78]
30	Cr(VI)	DAMP-Cu NCs	357/428	turn off	0–150 μM	8.5 μM	water	[24]
31	Cr(VI)	Thiosalicylic acid/Cysteamine-Cu NCs	355/411	turn off	0.1–1000 μM	30 nM	water	[21]
32	Cr(VI)	Cysteamine-Au/Cu NCs	350/436	turn off	0.2–100 μM	80 nM	water and human urine sample	[23]
33	Cr(Ⅵ)	Cu NCs@TA	360/430	turn off	0.03–60 µM	5 nM	water sample	[90]
34	Cu^2+^	D-Penicillamine -Cu NCs	391/673	turn on	0.95–6.35 ppm	0.3 ppm	tap water	[91]
35	Cu^2+^	CdSe QDs @ hPEI-Cu NCs	380/495,625	ratiometric	0.022–8.8 μM	8.9 nM	river water	[81]
36	Cu^2+^	GSH- Cu NCs	330/615	turn on	0.25–10 μM	170 nM	chalcocite	[38]
37	Cu^2+^	DNA-Cu/Ag NCs	480/576	turn on	5–200 nM	2.7 nM	soil and pond water	[42]
38	Cu^2+^	Cytidine-Cu NCs	300/380	turn on	0.05–2.0 µM	32 nM	lake water	[58]
39	Cu^2+^	BSA-Cu NCs	340/420	turn off	0.02–34 μM	1 nM	tap water	[20]
40	Cu^2+^	BSA-Cu NCs/ BSA-Au NCs	365/398,616	ratiometric	0.1–1 µM and 1–5 µM	23.4 nM	Tap, mineral, lake water	[87]

**Table 3:** List of fluorescent probes based on Cu NCs for sulfide anions detection.

No	Analytes	Sensors	Ex./Em. Maxima (nm)	Sensing Mechanism	Linear Range	Limit of Detection (LOD)	Real Sample	Ref.
1	H_2_S	PSS-PA-Cu NCs	325/655	turn-off	2–10 μM	650 nM	spring water	[94]
2	S^2^^−^	G-R-Cu NCs	400/490, 610	ratiometric	0.1–10 μM and 0.1–10 mM;	100 nM	chicken blood	[93]
3	S^2^^−^	TA-Cu NCs	360/441	turn-off colorimetric	0.7–80 μM 6–130 μM	0.1 μM 2.0 μM	natural water	[95]
4	S^2^^−^	Cu2+@MPA-Cu NCs	350/610	turn-off	0−600 μM	26.3 nM	food additives	[92]
5	S^2^^−^ and H_2_S	Cu NCs-CQD	365/469,622	ratiometric	26–128 nM	4.3 nM	—	[96]
6	S^2−^	Cu NCs	326/422	turn-on	5–110 μM	0.286 μM	tap water and river water	[10]

**Table 4:** List of fluorescent probes based on Cu NCs for detection of other analytes.

No	Analytes	Sensors	Ex./Em. Maxima (nm)	Sensing Mechanism	Linear Range	Limit of Detection (LOD)	Real Sample	Ref.
1	picric acid	Cu NCs-CA	393/480	turn-off	1–80 μM	0.14 μM	tap water, lake water and river water	[22]
2	trinitrophenol	DNA-Cu NCs	340/627	turn-off	0.1–100 μM	0.03 μM	water samples	[44]
3	picric acid	PM-GSH-Cu NCs	360/625	turn-off	9.9–43 μM	2.74 μM	water and matchstick	[37]
4	picric acid	Cys–Cu NCs	370/494	turn-off	2.5–25 mM	0.19 mM	tap and lake water	[100]
5	trinitrotoluene	CuNC/ZIF-8	365/600	turn-off	5–80 μM	8.5 μM	tap water	[36]
6	picric acid	Cu NCs	350/430	turn-off	2–40 mM	0.98 mM	water samples	[101]
7	quinoline yellow	L-Cys-Cu NCs	380/422,617	ratiometric	0.2–5.5 µM	110 nM	candies and soft drink	[97]
8	furazolidone	GSH-Cu NCs	366/426	turn off	0.05–60 µM	12 nM	aqueous sample	[98]
9	DPA	GSH-Cu NCs	380/422,617	ratiometric	0–20 µM	8 nM	aqueous sample	[99]
10	quinolones	Cys-Cu NCs	368/475	turn on	0.5–40 µM	8 nM	tablets	[102]
11	Sudan dyes Ⅰ Sudan dyes Ⅱ Sudan dyes Ⅲ Sudan dyes Ⅳ	PEI-Cu NCs	355/480	turn off	0.1–30 µM 0.1–30 µM 0.1–25 µM 0.1–25 µM	65 nM 70 nM 45 nM 50 nM	chilli powder sample	[103]
12	Bisphenol A	BSA-Cu NCs	chemiluminescence	turn off	0.001–10 µM	0.12 nM	water sample	[104]
13	Melamine	T30-Cu NCs	345/598	turn on	0.1–6 µM	95 nM	Milk	[105]

## Data Availability

No new data were created or analyzed in this study. Data sharing is not applicable to this article.
